# Prevalence of Mental Illness among Homeless People in Hong Kong

**DOI:** 10.1371/journal.pone.0140940

**Published:** 2015-10-20

**Authors:** Larina Chi-Lap Yim, Henry Chi-Ming Leung, Wai Chi Chan, Marco Ho-Bun Lam, Vivian Wai-Man Lim

**Affiliations:** 1 Department of Psychiatry, Prince of Wales Hospital, Shatin, Hong Kong; 2 Department of Psychiatry, The University of Hong Kong, Pok Fu Lam, Hong Kong; National Center of Neurology and Psychiatry, JAPAN

## Abstract

**Metholodogy:**

This study examined the prevalence and correlates of mental illness in homeless people in Hong Kong and explored the barriers preventing their access to health care. Ninety-seven Cantonese-speaking Chinese who were homeless during the study period were selected at random from the records of the three organisations serving the homeless population. The response rate was 69%. Seventeen subjects could not give valid consent due to their poor mental state, so their responses were excluded from the data analysis. A psychiatrist administered the Structured Clinical Interview for DSM-IV Axis-I disorders (SCID-I) and the Mini -Mental State Examination. Consensus diagnoses for subjects who could not complete the SCID-I were established by three independent psychiatrists.

**Findings:**

The point prevalence of mental illness was 56%. Seventy-one percent of the subjects had a lifetime history of mental illness, 30% had a mood disorder, 25% had an alcohol use disorder, 25% had a substance use disorder, 10% had a psychotic disorder, 10% had an anxiety disorder and 6% had dementia. Forty-one percent of the subjects with mental illness had undergone a previous psychiatric assessment. Only 13% of the subjects with mental illness were receiving psychiatric care at the time of interview. The prevalence of psychotic disorders, dementia and the rate of under treatment are hugely underestimated, as a significant proportion (18%) of the subjects initially selected were too ill to give consent to join the study.

**Conclusion:**

The low treatment rate and the presence of this severely ill and unreached group of homeless people reflect the fact that the current mode of service delivery is failing to support the most severely ill homeless individuals.

## Introduction

### Prevalence of Homelessness

Homelessness is a global problem. A report from the United Kingdom found that around 300,000 individuals were homeless at any given time [1] and recent data showed that an estimated 636,017 people (21 per 10,000 in the general population) experienced homelessness in the United States on any given night [2].

In Hong Kong, the number of registered street sleepers in the official record of the Social Welfare Department at the end of March, 2011 was 414 [3]. A local study estimated that there were 700 homeless people in Hong Kong [4].

### Mortality and Morbidity Associated with Homelessness

Homeless people have higher morbidity and mortality rates than the general population. In a review article [5], Scott concluded that they had a higher rate of physical morbidities. D’Amore, Hung, Chiang and Goldfrank [6] reported that homeless people who attended an emergency department had significantly higher rates of infectious disease. A survey conducted in Japan between1982 and 1991 [7] revealed that the prevalence of pulmonary tuberculosis in homeless people was around 20 times higher than in the general population. In a cohort of 6,308 homeless people in Philadelphia, Hibbs et al. [8] found that the age-standardised mortality rate was 3.5 times higher than in the general population, and that homeless people died at an earlier age. In another cohort study conducted in Denmark (N = 32711), Nielsen, Hjorthøj, Erlangsen and Nordentoft [9] found that the standardised mortality ratios for homeless men and women were 5.6 and 6.7 respectively. Life expectancy was lower than that of the general population. Registered substance abuse disorder was associated with higher mortality. A study that sampled 1,260 homeless people from shelters [10] showed that the age-adjusted mortality rates were four times higher than in the general population in the United States. Among homeless men, incarceration, prior use of injectable drugs and chronic homelessness increased the likelihood of death. Studies [11, 12] investigating the causes of death of homeless people have revealed that the homicide rate is high.

### Factors Associated with Homelessness

Homelessness may be caused by a constellation of factors including social influences, personal life experiences, personal vulnerability and mental health issues.

#### Social influences

A report from England [13] suggested that structural factors such as a reduced supply of affordable housing and a decline in the availability of social housing leads to homelessness.

#### Personal life experiences

Other factors leading to homelessness include personal issues, such as mortgage and rent arrears, breakdown of relationships and termination of short-hold tenancies [13]. Studies have also shown that childhood family disorganisation [14, 15], childhood abuse [16, 17] and lack of parental care [17] are associated with homelessness in adulthood.

#### Drugs and alcohol abuse

Studies using various different sampling frames have concluded that substance and alcohol use is associated with homelessness [18–22].

#### Deinstitutionalization

It has been suggested that deinstitutionalisation and a lack of community-based services for people with mental illness contribute to homelessness in the mentally ill [23–27]. Some authors [28, 29] have speculated that deinstitutionalisation might increase the rate of criminal behavior among homeless people and hence this group may be overrepresented in jails. In contrast, Cohen and Thompson [30] proposed that deinstitutionalisation might not contribute significantly to homelessness. After examining studies that explored the temporal relationship between socioeconomic changes, deinstitutionalisation and rates of homelessness, Cohen and Thompson argued that social, political and economic changes and a lack of low-income housing might be more important contributing factors.

### Mental Illness and Homelessness

In a study involving14, 888 adults, Shelton et al. [15] found that the presence of mental health problems was a significant independent risk factor for homelessness. Many studies have revealed high rates of mental illness among homeless people. A systematic review by Fazel, Khosla, Doll and Geddes [31] found that the most common mental disorders in the homeless population were alcohol dependence (8.1% to 58.5%) and drug dependence (4.5% to 54.2%). The prevalence of psychotic illness and depression ranged from 2.8% to 42.3%. The prevalence of personality disorder varied widely across studies, from 2% to 71%. Scott [5] found that 30% to 50% of homeless people had a mental illness and comorbidity of substance abuse and mental illness occurred in 20% of the homeless population. In Nielsen et al.’s [9] nationwide cohort study, 62.4% of men and 58.2% of women in the Danish homeless population had psychiatric disorders. Almost 36% had substance abuse disorders, and around 4% had disorders on the schizophrenia spectrum. A state-wide survey (N = 4730) [32] found that almost half of the formerly -homeless group had a one-year psychiatric diagnosis—a rate nearly twice that of the never -homeless group. The prevalence of alcohol use disorder comorbid with one or more psychiatric disorders was 15.1% in the formerly -homeless group—five times higher than the rate in the never -homeless group. Folsom and Jeste [33] found that schizophrenia was overrepresented in the homeless population, with a prevalence of from 4% to 16%. A study [34] that sampled 7,224 mentally ill homeless people from a multi-site outreach programme found high rates of 30-day suicidal ideation (37.5%) and suicide attempts (7.9%). Mentally ill homeless people have also been found to be more likely to be victims of physical assault, criminal activities and sexual harassment [35–38].

The majority of studies on mental illness and homelessness were conducted in Western countries. There is a paucity of data on the prevalence and correlates of mental illness in homeless people in Asia. The only published study sampled subjects from homeless shelters in Korea [39]. The lifetime prevalence of serious mental disorder was 67% and the lifetime prevalence of substance use disorder (53.8%), mood disorder (34.2%) and psychotic disorder (3.7%) was much higher than in the general population in Korea.

### Challenges in Research Related to Homelessness and Mental Illness

The results from previous studies on mental illness and homelessness are heterogeneous, making cross-country and cross-study comparison difficult. The adoption of such data for planning local services is therefore inappropriate. In addition, caution is needed when interpreting the findings from previous research in light of several methodological caveats as outlined below

#### Difficulties in estimating the size of the homeless population

Obtaining an accurate estimate of the size of the homeless population has always been challenging. Several studies [40–42] have discussed the practical difficulties in counting the homeless population. Iachan and Dennis [40] demonstrated that different geographical and temporal sampling methods evaluated different homeless populations. The National Coalition for the Homeless [42] criticised the ‘point-in-time count’, a commonly used method for counting the homeless population, because it misrepresents the magnitude and nature of homelessness. The point-in-time count method counts all individuals who are literally homeless on a given day or during a given week. This method dose not identify intermittently homeless individuals, and therefore tends to overestimate the proportion of people who are chronically homeless. A number of homeless individuals might be missed if they are not in the places easily accessed by researchers.

#### No uniform definition of homelessness

There is no uniform definition of homelessness. Scott [5] detailed several concepts and classifications of homelessness. ‘Homelessness’ can be conceptualised as ‘disaffiliation and detachment from society’, ‘any single person with no home of his own’ and ‘anyone who lacks adequate shelter, resources and community ties’. Homelessness can also be classified according to temporal, geographical and topographical features. Fischer and Breakey [43] proposed that the line between the truly homeless and sporadically or marginally housed people is blurred. The conventional definition, based on the lack of a permanent place to live, has led investigators to sample from streets and from various facilities that serve the needs of people without shelter. A broader definition, which encompasses marginally housed people and people at risk of homelessness, can include single room occupancy hostels, doubled-up accommodation and institutions such as jails, residential substance abuse programmes and hospitals as sampling sites. However, studies that adopted an operational definition of homelessness based on target facilities have often been criticised for representing only the higher functioning group of the diverse population of homeless individuals. In a systemic review by Fazel et al. [31], most of the reviewed articles sampled subjects from shelters or hostels. Of the 31 articles reviewed, only 5 featured subjects from the streets. Gill, Meltzer and Hinds [44] demonstrated that the prevalence of mental illness differed between samples from hostels, private-sector leased accommodation, night shelters and streets.

#### Miscellaneous challenges

Other problems underlying research related to mental illness and homelessness, which influence estimates of prevalence, include a lack of standardised diagnostic instruments, infrequent use of clinical examination, different abilities to detect true cases when screening instruments are used and a lack of consistent temporal reporting frames [40, 43]. Hwang [45] noted that most cross-sectional studies were biased towards overestimating the prevalence of mental illness because the duration of homelessness tends to be longer in people with mental illness. In a cross-sampling of shelter users, an individual who remains homeless for a longer period has a higher chance of being selected than one with a shorter period of homelessness.

### Homelessness in Hong Kong

#### Services for homeless people in Hong Kong

The Hong Kong government uses the notion of ‘street sleeper’ to conceptualise homelessness. The Social Welfare Department subsidises three non-government organisations (NGOs) to take care of street sleepers [3]. These organisations, namely the Salvation Army, St. James’ Settlement and the Christian Concern for the Homeless Association, each operate an integrated services team for street sleepers. Each NGO serves a specified area and the service scope covers the entire region of Hong Kong. The services they provide include counselling, night outreach, group activities, personal care such as bathing and hair-cutting, employment guidance, emergency financial assistance, emergency shelter, temporary hostel placement and referral to medical services [46]. There are other charitable organisations that serve homeless people in Hong Kong, but only the three NGOs mentioned above have full-time workers who provide a regular night outreach service.

#### Registration of homeless people in Hong Kong

The Social Welfare Department has set up a computerised street sleepers’ registry to record street sleepers’ personal data and the services they receive. Each NGO must record information about new street sleepers every month. The names of the street sleepers are removed from the list once they find alternative accommodation. The number of registered street sleepers at the end of March, 2011 was 414 [3].

The official list of the Social Welfare Department is not comprehensive. It is not a compulsory requirement for street sleepers to register with the Social Welfare Department. A report by the Society for Community Organization [47] considered why such a registry is unable to correctly estimate the number of street sleepers in Hong Kong. According to the criteria set by the Social Welfare Department, an individual has to sleep at the same place on seven consecutive days to be included in the register. The street sleepers are required to provide detailed personal information such as their history of homelessness and reasons for becoming homeless, many street sleepers therefore refuse to be registered. Once accommodation assistance is provided to a street sleeper, the name of the individual is removed from the registry in that month. This practice does not take the dynamic nature of homelessness into account. Some people leave hostels or shelters within a month and become street sleepers again. Despite the availability of rented accommodation, some individuals may sleep in the streets at night sporadically. For example, some may choose to sleep in the streets when the weather is too hot. Some have to sleep in public places after arguing with their family. Some may choose to sleep at a place that is near to their work place to save public transport fees.

The registry of the Social Welfare Department is not representative of homeless people in Hong Kong, as it excludes the groups who do not sleep at a fixed place at night, the short-term homeless who have not slept on the street for seven consecutive days, and those who refuse to complete the registration procedure. The most comprehensive list of homeless people in Hong Kong comes from the records of the three NGOs, each serving in a specified area in Hong Kong with no overlap between them.

The NGOs keep the records of all homeless individuals they have identified, disregarding the duration of homelessness and willingness to accept services. These records indicate that homeless people were identified from a wide range of sources. For example, some homeless people presenting themselves at the offices of NGOs and ask for assistance. Others are identified through regular night outreach. Local residents also report new street sleepers within their area to social workers of NGOs, as do other homeless people.

#### Local study on mental illness and homelessness in Hong Kong

There is a dearth of information about mental illness in the homeless population in Hong Kong. There is also a lack of mental health policy and services targeting this group. The only study of mentally ill homeless people was conducted by two NGOs in 2009 [4]. The study defined ‘homelessness’ as anyone sleeping in public places at night. Investigators identified 404 homeless clients, or their sleeping place, and 212 of them were interviewed. Based on the Brief Symptom Inventory, a self-report assessment tool, 62.7% of the subjects were suspected to have mental illness. Eleven percent of the subjects had previously received psychiatric assessment, 5% had attended psychiatric outpatient clinics in the previous six months, and one subject had been visited by the community psychiatric team.

The wave of deinstitutionalization has little impact on the psychiatric services in Hong Kong. In fact, the number of psychiatric beds has increased over the years. Since data on mental illness and homelessness in Hong Kong is lacking, how the increase in psychiatric beds affect the mentally ill homeless people is unknown.

#### Mental Health Services for Homeless People in Hong Kong

The findings of the aforementioned report, and the discrepancy between the limited services and the pressing needs of mentally ill homeless people, were brought up at a meeting at the Legislative Council in May, 2011 [48]. Representatives from NGOs highlighted the lack of mental health services for mentally ill homeless people. It was stated that homeless people who were suspected to be mentally ill could have an assessment at a place that was considered safe by all parties involved. This implied that outreach services would not be provided at all, as it was not possible to identify a safe place which was agreeable to all parties concerned. Representatives from NGOs raised the issue that there was no designated organisation to assess street sleepers with suspected mental illness. After this meeting, the NGOs were notified that the community psychiatric team from public hospitals would assess street sleepers in an outdoor location, provided that the street sleepers could be located and medical personnel were accompanied by social workers during the assessment.

## Objective

The aim of this study was to determine the prevalence of mental illness in the homeless population in Hong Kong and explore their barriers to accessing mental health services.

## Methodology

This was a cross-sectional prevalence study. There is no universal consensus on the definition of homelessness. For the current study, ‘a homeless person’ was defined as a person who had slept in a public place, street, shelter, abandoned building, or places not intended to be dwellings. This definition takes account of the dynamic nature of homelessness, results of local studies and government policy. As the duration of homelessness was not specified, the sporadically homeless group was also included as subjects.

Guided by local data [4], assuming a total of 700 homeless people and a prevalence of mental illness of 50%, a sample of 85 subjects was required to obtain a 95% confidence interval [49]. Potential subjects were randomly drawn from the lists of homeless clients at the three NGOs designated by the Social Welfare Department to provide services for homeless individuals across the whole territory of Hong Kong. Target subjects were invited for interview through the NGOs. Most interviews were conducted outside office hours and all were conducted in person by the principal investigator (PI), a psychiatrist with six years’ training, in the presence of social workers who provided liaison. Written informed consent was obtained for participation in the study and permission was given to trace past contact history within the local public health care system. The consent process was carried out the in presence of a social worker, who signed on the consent document as a witness. For subjects who refused to give written consent, oral consent was obtained, in the presence of a social worker who provided liaison. The social worker also signed on the consent document as a witness when the subject refused to provide written consent. Upon completion of the interview, a 30 HKD food coupon was offered to the study subject as a token of appreciation.

Chinese ethnicity Cantonese-speaking homeless individuals, as defined above, were identified as potential subjects. Individuals with impaired consciousness or inability to give valid consent were excluded from the study. Current psychiatric diagnoses and lifetime psychiatric diagnoses were obtained through the Chinese-bilingual Structured Clinical Interview for DSM-IV Axis I Disorders Patient Research Version (SCID-I). The electronic record of the public health care system was used to check for the presence of psychiatric diagnoses that could not be obtained through the SCID-I (dementia and intellectual disability). Other assessment tools included the Chinese version of the Mini Mental State Examination (MMSE), the GAF (Global Assessment of Functioning) [50] and a semi-structured questionnaire for demographic data and clinical history. Details of the mental state examination and background information were recorded for subjects who could not complete the SCID-I. The information was subsequently reviewed by three independent psychiatrists to generate a consensus DSM-IV diagnosis. Approval was obtained from the Joint Chinese University of Hong Kong New Territories East Cluster Clinical Research Ethics Committee. The study was conducted between October 2011 and June 2012.

### Statistical Analysis

The Statistical Package for the Social Sciences version 19 was used for statistical analysis. Chi square or Fisher’s exact tests were used to examine group difference for independent binary variables. The Student’s *t*-test was used for continuous variables. The Mann Whitney U test was used for non-normally distributed data. The level of statistical significance was set at 0.05.

## Results

**Fig 1:**
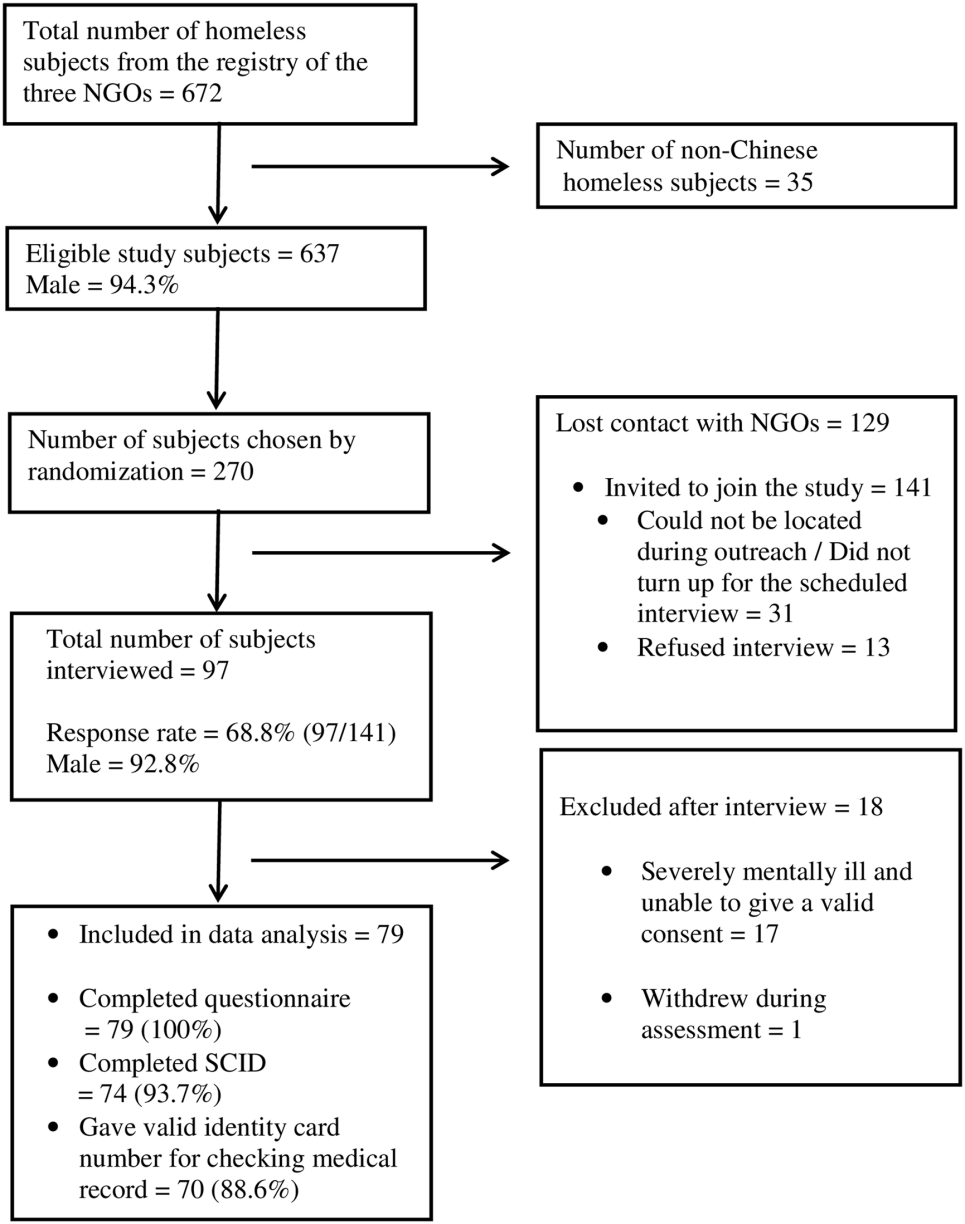
Subject Recruitment Process.

Attempts were made to approach 270 homeless individuals chosen by systematic randomization (Fig 1). Of these, 129 were no longer traceable. Social workers invited 141 (270–129) homeless individuals to join the study. Among the 141 invited individuals, 13 refused to participate in the study and 31 agreed initially but did not turn up for interview. A total of 97 subjects were interviewed (a response rate of 68.8%). One of the 97 subjects withdrew from the study. Seventeen subjects lacked the ability to give valid consent, due to underlying mental illness or cognitive impairment. Data for the remaining 79 subjects (81.4% of the interviewed subjects) were subjected to analysis.

### Demographics and Characteristics of the Subjects

As shown in Table 1, the majority (94%) of subjects were male. Around 70% were above 50 years of age. Just less than half (47%) had attained secondary education or above. A quarter (24.1%) claimed to be married, 42% were single and 29% were divorced. A third (32.9%) of the sample was employed at the time of interview and 89% were receiving financial assistance from the government.

**Table 1:** Demographics and homeless history of the study subjects (N = 79).

**Age group**	
21 to 35	7
36 to 50	17
51 to 65	43
66 to 80	10
Older than 80	2
**Sex**,	
male, *n* (%)	74 (93.7)
**Education level**	
Primary education or below, *n* (%)	42 (53.2)
Secondary education or above, *n* (%)	37 (46.8)
**Marital status**	
Married, *n* (%)	19 (24.1)
Single, *n* (%)	33 (41.8)
Divorced, *n* (%)	23 (29.1)
Widowed, *n* (%)	3 (3.8)
**Employed, *n* (%)**	26 (32.9)
**Duration of unemployment**	
≤1 year, *n* (%)	12 (15.2)
2–5 years, *n* (%)	15 (19.0)
6–10 years, *n* (%)	11 (13.9)
>10 years, *n* (%)	12 (15.2)
Never worked, *n* (%)	2 (2.5)
Unsure, *n* (%)	1 (1.3)
**No regular financial support, *n* (%)**	11 (13.9)
**Age of first homelessness, years (*M*, *SD*)**	41.6 ± 15.5 (range: 8–79)
**Total duration of homelessness**	
≤ 1 year, *n* (%)	14 (17.7)
2–5 years, *n* (%)	27 (34.2)
6–10 years, *n* (%)	9 (11.4)
>10 years, *n* (%)	13 (16.5)
Forgot, *n* (%)	16 (20.3)
**With repeated periods of homelessness, *n* (%)**	44 (55.7)
**Still homeless at the time of interview, *n* (%)**	59 (74.7)
**Sleep place**	
Outdoor, *n* (%)	56 (70.9)
Indoor, *n* (%)	23 (29.1)
Living/ lived in hostel for homeless people, *n* (%)	23 (29.1)
**Reasons for becoming homeless, *n* (%)**	
Financial problem	56 (70.9)
Interpersonal relationship difficulty	24 (30.4)
Disliked the environment or regulations in hostel/ rented place	28 (35.4)

*Notes*. *M* represents mean. *SD*represents standard deviation.

The mean age at first homelessness was 41.6 ± 15.5 (range: 8–79). More than 80% of the subjects had been homeless for more than a year. Half (56%) had experienced repeated periods of homelessness and only 30% had lived in hostels for the homeless before. Seventy-five per cent were homeless at the time of interview. Most of them (71%) slept in an outdoor location. A financial problem was the most commonly reported reason for becoming homeless, followed by dissatisfaction with the environment or regulations of hostels or rented places.

#### Forensic history

As reported in Table 2, almost half (49%) of the subjects had a forensic record. One fifth (20%) had a history of drug-related crime and a further fifth (22%) had a history of violent- crime. One third (32%) had a history of theft or robbery.

**Table 2:** Self-reported Forensic History (N = 79).

	*n* (%)
**Number of subjects with forensic record**	39 (49.4)
**Forensic history**	
Drug-related crime	16 (20.3)
Theft/ Robbery	25 (31.6)
Violence	17 (21.5)
Keeper of a brothel	3 (3.8)
Sold pirated goods	3 (3.8)
Facilitated illegal immigration	1 (1.3)
Criminal intimidation	1 (1.3)
Loitering	1 (1.3)
Making forged document	1 (1.3)
Communal gambling	2 (2.5)
Refused to tell	1 (1.3)

#### Medical history

There was a high prevalence of past history of tuberculosis in the study subjects. Skin and subcutaneous tissue infection was the most commonly recorded medical problem, as shown in Table 3. More than half of the subjects who had physical complaints at the time of interview did not seek medical attention. More than half of the group who did not seek help perceived that it was not necessary to consult a doctor for their symptoms.

**Table 3:** Medical History, Current Physical Complaints and Reasons for Not Seeking Medical Attention (N = 79).

**Most commonly reported medical history based on the public health care records and self-report**	*n* (%)
Skin infection/ abscess/ wound infection	31 (39.2)
Loss of consciousness	14 (17.7)
Gastrointestinal bleeding/ gastritis/ gastric ulcer/ duodenal ulcer	11 (13.9)
Assaulted by others leading to AED admission	10 (12.7)
Hypertension	10 (12.7)
Alcohol-related injury or illness	9 (11.4)
Chest infection or COPD requiring in-patient treatment	9 (11.4)
Tuberculosis	8 (10.1)
Pneumothorax	6 (7.6)
**Number of subjects with physical complaints**	**51 (64.6)**
**Most commonly reported physical complaints at interview**	
Musculoskeletal system	20 (25.3)
Oral/ dental	13 (16.5)
Gastrointestinal	9 (11.4)
Respiratory	9 (11.4)
Skin	7 (8.9)
**Had consulted a doctor for the physical complaints (*n* = 51)**	**23 (45.1)**
**Most commonly reported reasons for not seeking medical advice/ attending follow-up (*n* = 28)**	
Perceived no need to seek medical attention/ self- medicated/ self-help methods	15 (53.6)
Financial difficulty	11 (39.3)
Inconvenient appointment time/ long waiting time/ clinics too far away	10 (35.7)
Didn’t know how or where to seek help	6 (21.4)

### Psychiatric history

Forty-six per cent of the subjects reported that they had experienced mental health problems in the past (Table 4). Only a third (36%) of them had sought medical attention. The majority of those who did not seek help either perceived it as unnecessary or preferred solving the problem by themselves. Fifteen per cent of subjects reported a history of suicide attempt.

**Table 4:** Self-reported Psychiatric history (N = 79).

	*n* (%)
**Had problems with mental health in the past**	36 (45.6)
**Type of self-reported mental health problems**	
Mood problem/ poor temper control	19 (24.1)
Suicidal ideation	6 (7.6)
Psychotic symptoms	6 (7.6)
Substance misuse/ dependence	6 (7.6)
Anxiety symptoms	3 (3.8)
Homicidal idea	1 (1.3)
Sleep problem	1 (1.3)
Did not give details	7 (8.9)
**History of suicidal attempt**	12 (15.2)
**Had sought medical advice for the reported mental health problems (*n* = 36)**	13 (36.1)
**Commonly reported reasons for not seeking medical advice (*n* = 23)**	
Perceived that it was not necessary to seek medical attention/ It was not mental illness/ It was a self-remitting condition	16 (69.6)
Preferred self-help	7 (30.4)
Perceived that doctors won’t be able to help	4 (17.4)

#### Comparing subjects with and without a previous suicide attempt

Subjects with a history of suicide attempt had a higher rate of mood disorders and substance-related disorders (Table 5). There was no significant difference in health-seeking behaviour between those who had attempted suicide and those who had not.

**Table 5:** Comparison of Subjects with Suicide Attempt (*n* = 12) and without Suicide Attempt (*n* = 67).

	History of suicide attempt (*n* = 12)	No history of suicide attempt (*n* = 67)	*p* value
Mood disorders, *n* (%)	7 (58.3)	17 (25.4)	.038*
Anxiety disorders, *n* (%)	2 (16.7)	6 (9.0)	.600
Psychotic disorders, *n* (%)	3 (25.0)	5 (7.5)	.098
Alcohol-related disorders, *n* (%)	2 (16.7)	18 (26.9)	.720
Substance-related disorders, *n* (%)	8 (66.7)	12 (17.9)	.001**
Did not seek medical attention OR Did not attend follow-up regarding mental health problems, *n* (%) ^1^	8 (88.9)	24 (88.9)	1.000

“*” indicates a *p* value <.05.

“**” indicates a *p* value <.01.

^**1**^ *n* = 36 (36 out of 79 subjects reported that they had mental health problems in the past).

#### Prevalence of mental illness

The prevalence of mental illness in the subjects is reported in Table 6. Combining the diagnoses generated by the SCID with consensus diagnoses by independent psychiatrists, 56% of the subjects had a current diagnosis of mental illness at the time of interview. The estimated lifetime prevalence of mental illness was 71%. Thirty percent of the subjects had a lifetime history of mood disorder, 25% had an alcohol use disorder, 25% had a substance use disorder, 10% had a psychotic disorder, 10% had an anxiety disorder and 6% had dementia. Twenty-three per cent had two or more psychiatric diagnoses (Table 7).

**Table 6:** Prevalence of Mental Illness (N = 79).

	*n* (%)
Presence of any mental illness	56 (70.9)
Subjects with psychiatric diagnosis in the past month	44 (55.7)
**Mood disorders**	**24 (30.4)**
Past major depressive disorder	8
Current major depressive disorder	5
With psychotic features	2
Without psychotic features	3
Dysthymia	15
Depressive disorder NOS	3
Bipolar disorder	1
**Alcohol abuse/ dependence**	**20 (25.3)**
Past alcohol abuse/ dependence	8
Current alcohol dependence	8
Current alcohol abuse	4
**Substance abuse/dependence, substance-induced mood, anxiety, or psychotic disorder**	**20 (25.3)**
Past history of substance abuse/ dependence	5
Opiate abuse/ dependence	18 (22.8)
Past history of opiate dependence	3
Current opiate dependence	15
Current sedative/ amphetamine/ cough mixture/ cocaine/ cannabis dependence	11
Current substance-induced psychotic disorder	4
Current substance-induced mood disorder	2
Current substance-induced anxiety disorder	1
**Psychotic disorders**	**8 (10.1)**
Schizophrenia	3
Schizoaffective	1
Psychotic disorder NOS	4
**Anxiety disorders**	**8 (10.1)**
Past history of obsessive compulsive disorder	2
Panic disorder	2
Specific phobia	2
Anxiety disorder NOS	1
Obsessive compulsive disorder	1
**Dementia**	**5 (6.3)**
**Mild grade intellectual disability**	**2 (2.5)**
**Two or more psychiatric comorbidities**	**18 (22.8)**

**Table 7:** Psychiatric Comorbidity (N = 79).

	n (%)
Comorbid mood disorder and alcohol/ drug abuse or dependence	13 (16.5)
Comorbid psychotic disorder and alcohol/ drug abuse or dependence	2 (2.5)
Comorbid alcohol abuse/ dependence and drug abuse/ dependence	6 (7.6)
Comorbid mood and psychotic disorders	2 (2.5)
Two or more psychiatric comorbidities	18 (22.8)

Seventy-one subjects completed the MMSE. The cutoff points for MMSE were the same as the optimal cutoffs in Chinese elderly, suggested by Chiu et al [51]. The cutoff was 18 for those with no education. The cutoff was 20 for those with one to two years of education. The cutoff was 22 for those with more than two years of education. Fifteen of them scored less than the cut-off point.

#### Treatment history

Forty-one percent of the mentally ill subjects had received a psychiatric assessment before the study and thirteen per cent of the mentally ill subjects were receiving psychiatric care at the time of interview (Table 8). Among the mentally ill subjects, there was no statistically significant difference between the groups with and without a previous psychiatric assessment (Table 9).

**Table 8:** History of Psychiatric Assessment and Treatment in Subjects with Mental Illness (*n* = 56).

	*n* (%)
**Had received assessment for their mental illness**	23 (41.1)
**Psychiatric diagnosis in subjects who had previous psychiatric assessment**	
Psychotic disorder	5
Substance abuse/ dependence disorder	10
Mood disorder	10
Alcohol abuse/ dependence disorder	4
Dementia	2
Mental retardation	2
Anxiety disorder	2
**Receiving psychiatric service during the study period**	7 (12.5)
**Psychiatric diagnosis in subjects who were receiving psychiatric service at the time of interview**	
Psychotic disorder	4
Mood disorder	3
Substance abuse/ dependence disorder	3
Dementia	1
Mental retardation	2
Alcohol use/ dependence disorder	1
Anxiety disorder	1

**Table 9:** Comparison of Mentally Ill subjects with and without Previous Psychiatric Assessment (*n* = 56).

	Had previous psychiatric assessment	No previous psychiatric assessment	*p* value
**Psychiatric diagnoses (*n*)**			
Mood disorder (24), *n* (%)	10 (50.0)	14 (38.9)	.574
Anxiety disorder (8), *n* (%)	2 (10.0)	6 (16.7)	.697
Psychotic disorder (8), *n* (%)	5 (25.0)	3 (8.3)	.118
Alcohol-related disorder (20), *n* (%)	4 (20.0)	16 (44.4)	.086
Substance-related disorder (20), *n* (%)	10 (50.0)	10 (27.8)	.146

#### Association between mental Illness, history of homelessness and demographic features

Mental illness vs. no mental illness (Table 10)

**Table 10:** Comparison of Subjects with Mental Illness (*n* = 56) and without Mental Illness (*n* = 23).

	No mental illness	History of mental illness	*p* value
	(*n* = 23)	(*n* = 56)	
**Demographic features**			
Age, years, (*M*, *SD)*	56.7 (11.5)	54.7 (12.6)	.498
Sex, male, *n* (%)	22 (95.7)	52 (92.9)	1.000
Primary education or below, *n* (%)	13 (56.5)	29 (51.8)	.811
Married, *n* (%)	5 (21.7)	14 (25.5)	.727
Employed, *n* (%)	8 (34.8)	18 (32.1)	.821
Duration of unemployment, months, median (*IQR*)	36 (123)	66 (141)	.386
Receiving financial assistance	18 (78.3)	50 (89.3)	.282
Last contact with relatives, months, *Md (IQR)*	36 (243)	3 (120)	.057
**Homelessness-related information**			
Age of first homelessness, years, (*M*, *SD)*	41.7 (17.7)	41.6 (14.6)	.977
Total duration of homelessness, months, median (*IQR*)	48 (48)	60 (96)	.293
Duration of current episode of homelessness, months, median (*IQR*)	36 (132)	42 (96)	.519
Repeated episodes of homelessness, n (%)	12 (52.2)	32 (57.1)	.686
Lived in homeless hostel before	7 (30.4)	16 (28.6)	1.000
**Self-reported reasons for becoming homeless**			
Financial problem	18 (78.3)	38 (67.9)	.355
Interpersonal relationship difficulty	5 (21.7)	19 (33.9)	.285
Disliked the environment/ regulations in hostel/ rented place	5 (21.7)	23 (41.1)	.103
Enjoyed the freedom of the homeless status	4 (17.4)	2 (3.6)	.056
**Presence of psychosocial stressors (self-report)**	12 (52.2)	38 (67.9)	.189
**Forensic history**			
Presence of forensic record, *n* (%)	8 (36.4)	31 (55.4)	.131
History of drug-related crime, *n* (%)	1 (4.5)	15 (26.8)	.031*
History of theft/ robbery, *n* (%)	6 (27.3)	19 (33.9)	.571
History of violence, *n* (%)	3 (13.6)	14 (25.0)	.368
**Suicide attempt**			
History of attempted suicide, *n* (%)	2 (8.7)	10 (17.9)	.492
**GAF score**			
GAF, *Md (IQR)*	59.9 (9.6)	59 (24)	.129

*Notes*. *M* represents mean. *SD* represents standard deviation. *Md* represents median. *IQR* represents interquartile range.

“*” indicates a *p* value <.05.

A higher frequency of drug-related crimes was found in subjects with a history of mental illness (*p* <.05).

Mood or anxiety disorders vs. no mental illness (Table 11)

**Table 11:** Comparison of Subjects with Mood or Anxiety disorder (*n* = 27) and Subjects without Mental Illness (*n* = 23).

	No mental illness	History of mood or anxiety disorder	*p* value
	(*n* = 23)	(*n* = 27)	
**Demographic features**			
Age, years, (*M*, *SD)*	56.7 (11.5)	53.0 (11.9)	.256
Sex, male, *n* (%)	22 (95.7)	24 (88.9)	.614
Primary education or below, *n* (%)	13 (56.5)	15 (55.6)	.945
Married, *n* (%)	5 (21.7)	6 (22.2)	.967
Employed, *n* (%)	8 (34.8)	10 (37.0)	.869
Duration of unemployment, months, median (*IQR*)	36 (123)	72 (96)	.483
**Homelessness-related information**			
Age of first homelessness, years, (*M*, *SD)*	41.7 (17.7)	44.2 (13.9)	.588
Total duration of homelessness, months, median (*IQR*)	48 (48)	24 (108)	.960
Duration of current episode of homelessness, months, median (*IQR*)	36 (132)	24 (57)	.715
Repeated episodes of homelessness, *n* (%)	12 (52.2)	13 (48.1)	.777
Lived in homeless hostel before, *n* (%)	7 (30.4)	9 (33.3)	.827
**Presence of psychosocial stressors (self-report)**	12 (52.2)	22 (81.5)	.027*
**Forensic history**			
Presence of forensic record, *n* (%)	8 (36.4)	14 (51.9)	.278
History of drug-related crime, *n* (%)	1 (4.5)	8 (29.6)	.030*
History of theft/ robbery, *n* (%)	6 (27.3)	10 (37.0)	.468
History of violence, *n* (%)	3 (13.6)	5 (18.5)	.715
**Suicide attempt**			
History of attempted suicide, *n* (%)	2 (8.7)	7 (25.9)	.152
**GAF score**			
GAF, (*M*, *SD)*	59.9 (9.6)	58.2 (13.7)	.596

*Notes*. *M* represents mean. *SD* represents standard deviation. *Md* represents median. *IQR* represents interquartile range.

“*” indicates a *p* value <.05.

Subjects with mood or anxiety disorders were more likely to self-report psychosocial stressors (*p* < .05) and have drug-related crimes (*p* < .05).

Psychotic disorders vs. no mental illness (Table 12)

**Table 12:** Comparison of Subjects with Psychotic Disorder (*n* = 8) and Subjects without Mental Illness (*n* = 23).

	No mental illness	History of psychotic disorder	*p* value
	(*n* = 23)	(*n* = 8)	
**Demographic features**			
Age, years, (*M*, *SD)*	56.7 (11.5)	47.4 (12.2)	.060
Sex, male, *n* (%)	22 (95.7)	8 (100)	1.00
Primary education or below, *n* (%)	13 (56.5)	1 (12.5)	.045*
Married, *n* (%)	5 (21.7)	0 (0)	.291
Employed, *n* (%)	8 (34.8)	2 (25.0)	1.00
Duration of unemployment, months, median (*IQR*)	36 (123)	72 (140)	.841
**Homelessness-related information**			
Age of first homelessness, years, (*M*, *SD)*	41.7 (17.7)	37.0 (9.09)	.512
Total duration of homelessness, months, median (*IQR*)	48 (48)	54 (129)	.367
Duration of current episode of homelessness, months, median (*IQR*)	36 (132)	27 (135)	.665
Repeated episodes of homelessness, *n* (%)	12 (52.2)	6 (75.0)	.412
Lived in homeless hostels before, *n* (%)	7 (30.4)	5 (62.5)	.206
**Presence of psychosocial stressors (self-report)**	12 (52.2)	6 (75.0)	.412
**Forensic history**			
Presence of forensic record, *n* (%)	8 (36.4)	5 (62.5)	.242
History of drug-related crime, *n* (%)	1 (4.5)	2 (25.0)	.166
History of theft/ robbery, *n* (%)	6 (27.3)	3 (37.5)	.666
History of violence, *n* (%)	3 (13.6)	3 (37.5)	.300
**Suicide attempt**			
History of attempted suicide, *n* (%)	2 (8.7)	3 (37.5)	.093
**GAF score**			
GAF, (*M*, *SD)*	59.9 (9.6)	46.0 (13.3)	.003**

*Notes*. *M* represents mean. *SD* represents standard deviation. *Md* represents median. *IQR* represents interquartile range.

“*” indicates a *p* value <.05.

“**” indicates a *p* value <.01.

Subjects with psychotic disorders had significantly lower GAF score (*p* < .05).

Alcohol abuse/ dependence vs. no mental illness (Table 13)

**Table 13:** Comparison of Subjects with History of Alcohol Abuse/ Dependence (n = 20) and Subjects without Mental Illness (n = 23).

	No mental illness	History of alcohol abuse/ dependence	*p* value
	(*n* = 23)	(*n* = 20)	
**Demographic features**			
Age, years, (*M*, *SD)*	56.7 (11.5)	53.5 (7.8)	.286
Sex, male, *n* (%)	2 (95.7)	19 (95.0)	1.00
Primary education or below, *n* (%)	13 (56.5)	10 (50.0)	.669
Married, *n* (%)	5 (21.7)	5 (25.0)	1.00
Employment status, Employed, *n* (%)	8 (34.8)	9 (45.0)	.494
Duration of unemployment, months, median (*IQR*)	36 (123)	120 (204)	.395
**Homelessness-related information**			
First age of homelessness, years, (*M*, *SD)*	41.7 (17.7)	41.5 (9.6)	.958
Total duration of homelessness, months, median (*IQR*)	48 (48)	60 (72)	.284
Duration of current episode of homelessness, months, median (*IQR*)	36 (132)	60 (96)	.849
Repeated episodes of homelessness, *n* (%)	12 (52.2)	11 (55.0)	.853
Lived in homeless hostels before, *n* (%)	7 (30.4)	4 (20.0)	.434
**Presence of psychosocial stressors (self-report)**	12 (52.2)	14 (70.0)	.233
**Forensic history**			
Presence of forensic record, *n* (%)	8 (36.4)	13 (65.0)	.064
History of drug-related crime, *n* (%)	1 (4.5)	5 (25.0)	.087
History of theft/ Robbery, *n* (%)	6 (27.3)	9 (45.0)	.231
History of violence, *n* (%)	3 (13.6)	7 (35.0)	.152
**Suicide attempt**			
History of attempted suicide, *n* (%)	2 (8.7)	2 (10.0)	1.00
**GAF score**			
GAF, (*M*, *SD)*	59.9 (9.6)	58.5 (13.0)	.685

*Notes*. *M* represents mean. *SD* represents standard deviation. *Md* represents median. *IQR* represents interquartile range.

There was no statistically significant difference between subjects with a history of alcohol abuse or dependence and those with no mental illness.

Substance abuse/ dependence vs. no mental illness (Table 14)

**Table 14:** Comparison of Subjects with History of Substance Abuse/ Dependence (*n* = 20) and Subjects without Mental Illness (*n* = 23).

	No mental illness	History of substance abuse/ dependence	*p* value
	(*n* = 2 3)	(*n* = 20)	
**Demographic features**			
Age, years, (*M*, *SD)*	56.7 (11.5)	54.0 (8.6)	.386
Sex, male, *n* (%)	22 (95.7)	19 (95.0)	1.00
Primary education or below, *n* (%)	13 (56.5)	13 (56.0)	.571
Married, *n* (%)	5 (21.7)	5 (26.3)	1.00
Employed, *n* (%)	8 (34.8)	3 (15.0)	.138
Duration of unemployment, months, median (*IQR*)	36 (123)	30 (110)	.845
**Homelessness-related information**			
First age of homelessness, years, (*M*, *SD)*	41.7 (17.7)	35.6 (11.7)	.195
Total duration of homelessness, months, median (*IQR*)	48 (48)	120 (156)	.008**
Duration of current episode of homelessness, months, median (*IQR*)	36 (132)	48 (96)	.646
Repeated episodes of homelessness, *n* (%)	12 (52.2)	15 (75.0)	.122
Lived in homeless hostels before, *n* (%)	7 (30.4)	5 (25.0)	.692
**Presence of psychosocial stressors (self-report)**	12 (52.2)	13 (65.0)	.395
**Forensic history**			
Presence of forensic record, *n* (%)	8 (36.4)	19 (95.0)	<.001**
History of drug-related crime, *n* (%)	1 (4.5)	14 (70.0)	<.001**
History of theft/ robbery, *n* (%)	6 (27.3)	11 (55.0)	.067
History of violence, *n* (%)	3 (13.6)	10 (50.0)	.011*
**Suicide attempt**			
History of attempted suicide, *n* (%)	2 (8.7)	8 (40.0)	.028*
**GAF score**			
GAF, (*M*, *SD)*	59.9 (9.6)	52.4 (9.5)	.014*

*Notes*. *M* represents mean. *SD* represents standard deviation. *Md* represents median. *IQR* represents interquartile range.

“*” indicates a *p* value <.05.

“**” indicates a *p* value <.01.

Subjects with substance use disorders had a significantly longer total duration of homelessness (*p* < .01) and lower GAF score (*p* < .05). They were also more likely to have a forensic record (*p* < .01), a history of drug-related crime (*p* < .01), a history of violent crime (*p* < .05) and a history of attempted suicide (*p* < .05).

Two or more psychiatric comorbidities vs. no mental illness (Table 15)

**Table 15:** Comparison of Subjects with Two or More Psychiatric Comorbidities (*n* = 18) and Subjects without Mental Illness (*n* = 23).

	No mental illness	Two or more psychiatric comorbidities	*p* value
	(*n* = 23)	(*n* = 18)	
**Demographic features**			
Age, years, (*M*, *SD)*	56.7 (11.5)	53.3 (7.1)	.598
Sex, male, *n* (%)	22 (95.7)	17 (94.4)	1.000
Primary education or below, *n* (%)	13 (56.5)	10 (55.6)	.951
Married, *n* (%)	5 (21.7)	3 (16.7)	1.000
Employed, *n* (%)	8 (34.8)	6 (33.3)	.923
Duration of unemployment, months, median (*IQR*)	36 (123)	66 (96)	.583
Receiving financial assistance	18 (78.3)	17 (94.4)	.205
Last contact with relatives, months, *Md (IQR)*	36 (243)	6 (53)	.117
**Homelessness-related information**			
Age of first homelessness, years, (*M*, *SD)*	41.7 (17.7)	41.5 (8.8)	.967
Total duration of homelessness, months, median (*IQR*)	48 (48)	72 (120)	.203
Duration of current episode of homelessness, months, median (*IQR*)	36 (132)	36 (99)	1.000
Repeated episodes of homelessness, n (%)	12 (52.2)	11 (61.1)	.567
Lived in homeless hostel before	7 (30.4)	6 (33.3)	.843
**Presence of psychosocial stressors (self-report)**	12 (52.2)	15 (83.3)	.037*
**Forensic history**			
Presence of forensic record, *n* (%)	8 (36.4)	14 (77.8)	.009**
History of drug-related crime, *n* (%)	1 (4.5)	9 (50.0)	.002**
History of theft/ robbery, *n* (%)	6 (27.3)	8 (44.4)	.257
History of violence, *n* (%)	3 (13.6)	7 (38.9)	.140
**Suicide attempt**			
History of suicide, *n* (%)	2 (8.7)	6 (33.3)	.109
**GAF score**			
GAF, *Md (IQR)*	59.9 (9.6)	52.3 (12.7)	.034*

*Notes*. *M* represents mean. *SD* represents standard deviation. *Md* represents median. *IQR* represents interquartile range.

“*” indicates a *p* value <.05.

“**” indicates a *p* value <.01.

Subjects with two or more psychiatric comorbidities were more likely to report psychosocial stressors (*p* < .05), have a forensic record (*p* < .01), have a history of drug-related crimes (*p* < .01) and have a lower GAF score (*p* < .05).

### The Severely Ill Group Who Could not Complete the Assessments

Due to poor mental state, 17 subjects (18% of the 97 initially chosen subjects) failed to complete the questionnaire or SCID-I and were also unable to give consent to join the study. They were not included in the data analysis. Only descriptive accounts of their situation could be given. The majority of these subjects had extremely poor hygiene, florid psychotic symptoms and formal thought disorder. Cognitive impairment was evident in a number of subjects. Many were malnourished. None had contactable relatives. All of them slept at an outdoor location at night and most of them had not utilized services for homeless people. None had any known psychiatric contact history. None had been assessed by the community psychiatric team from the public hospital.

## Discussion

### Prevalence of Mental Illness in Homeless People in Hong Kong

The estimated prevalence of a lifetime history of mental illness was 71%. Fifty-six percent of the subjects had a psychiatric diagnosis at the time of interview. These figures were higher than those reported in overseas prevalence studies in the homeless [5, 9, 31–32].

The higher prevalence of mental illness in our study subjects might be due to the different sampling methods and definitions of homelessness used. As mentioned in the introduction, subjects in previous studies were usually recruited from shelters or facilities for the homeless, overlooking those sleeping rough on streets and those who did not use these facilities. Studies that used different sampling methods reported a higher rate of mental illness in samples drawn from the streets [44].

The reported prevalence of mental illness in this study is an underestimate. A significant proportion of the initially chosen subjects were not included in data analysis because they lacked the ability to give a valid consent due to underlying mental illness or cognitive impairment. The situation of this group of homeless people with serious mental illness has often gone unnoticed. Observational findings or reports about this group, whose mental illness renders them unable to complete the required assessment and interview, are lacking. In previous studies on mental illness and homelessness, most of the interviewers were not psychiatrists and hence psychiatric diagnoses were generated from standardised diagnostic instruments only. The above limitations have led to the underestimation of the prevalence of mental illness and the magnitude of the difficulties in this group. As a result, the need for proactive treatment might have been overlooked.

#### Mood and anxiety disorders

The prevalence of mood disorders and anxiety disorders in the study subjects was 30% and 10% respectively. Overseas studies have also reported high rates of depressive symptoms in homeless people [52–54]. A study [55] that compared scores on the Center for Epidemiological Studies Depression Scale showed that the percentage of homeless people who fit the criteria for clinical caseness was nearly four times greater than in the general population.

In the current study, there was no significant difference between the groups with mood or anxiety disorders and without mental illness in terms of functional level, as reflected by the GAF score (Table 11). More than a third of the subjects were employed at the time of interview. This group had a significantly higher frequency of self-reported psychosocial stressors. Over 80% of this group reported that they were distressed by financial difficulties, unemployment, conflicts with family, marital discord and housing problems. Irwin et al. [54] sampled over 1, 000 homeless subjects from soup kitchens, shelters and the street and found that a higher level of perceived social support and a higher education level lowered depressive symptomatology, whereas a higher frequency of stressful events and daily difficulties led to greater depressive symptoms. Schutt et al. [53] concluded that perceived social support lessened distress and suicidal thoughts in homeless people. In this regard, social work services and welfare assistance may be important factors for combating mental illness and homelessness.

Diagnosing depression in the homeless population is difficult [56–57]. Vázquez et al [56] suggested that depression has been a ‘silent disorder’ in the homeless population, which may prevent the sufferers from seeking help and utilising effective coping strategies. Seventeen per cent of the study subjects had comorbid mood disorders and alcohol or substance use disorders. Such comorbidity might mask the presence of depressive symptoms.

Coordinated, multidisciplinary support from social workers, mental health professionals and detoxification services will be important to help this group of homeless people.

#### Alcohol abuse or dependence

The lifetime prevalence of alcohol abuse or dependence was 25% in the study subjects. A review of 29 studies conducted worldwide estimated an alcohol dependence prevalence of 37.9% among homeless populations [31] and found that alcohol dependence was one of the most common mental disorders in homeless people. The relatively low rate of alcohol use disorders in the subjects in the current study could be due to a generally lower level of alcohol consumption in Hong Kong, compared with other countries [58].

In their review of the epidemiology of alcohol use in homeless people, Fischer et al. [43] found that homeless people who were alcohol-dependent had more severe patterns of drinking than non-homeless alcoholics. They had fewer friends and were more likely to have severed family relationships than homeless people who were not alcohol-dependent. The homeless alcoholics had more physical and psychiatric comorbidities, and were more likely to be involved in health-endangering behaviour, prostitution and criminal activities. A study that sampled 266 homeless people from shelters [59] found that 78% were drinking hazardously and 82% had cognitive impairment. The estimated prevalence of alcohol-related brain injury was 21%. With appropriate treatment, recovery from alcohol-related brain injury is possible [60]. In the present study, 11% of the subjects had received treatment in public hospitals for alcohol-related injury or illness. Medical professionals should seize the chance to refer social services and psychiatric services once they are in contact with the health care system. Given the high risk of comorbidities in homeless alcoholics, the use of the Mental Health Ordinance for compulsory treatment must be considered for those who are clearly incapable of independent living, uncooperative with treatment and have inadequate social support.

#### Substance abuse or dependence

Twenty-five percent of the study subjects had a history of substance abuse or dependence. This group had a longer total duration of homelessness, were more likely to have a forensic record, and had a higher frequency of drug-related crimes, violence-related crimes and suicide attempts. They also had lower GAF scores than those without mental illness. In Hong Kong, this subgroup of homeless people is usually managed by a range of service sectors, including the correctional service department, detoxification service units and the NGOs looking after the homeless population. Health care services are involved only when physical or psychiatric complications emerge. Homeless shelters and hostels in Hong Kong do not accept residents with active drug use. Detoxification centres require residents to be detoxified first. Hence, there has been a lack of concerted effort to provide holistic care for these needy clients. Joint efforts across the different service sectors might be more effective in treating this subgroup of homeless people.

#### Intellectual disability

The local electronic public health record showed that two of the subjects had mental retardation. The true prevalence of intellectual disability in our study subjects was not known, as the study did not include any formal intelligence assessment. Homeless people with intellectual disability are often overlooked by society and by researchers. A review of homelessness research in the United Kingdom from 1990 to 2000 [61] did not identify any study on intellectual disability and homelessness. Local reports and surveys offer no data for this group either. The lack of data concerning intellectually disabled homeless people reflects the inadequate attention paid to this vulnerable population, who are more susceptible to abuse.

#### Dementia and cognitive Impairment

Six per cent of the subjects received a diagnosis of dementia. Seventy-one subjects completed the MMSE and 21% had cognitive impairment. This prevalence rate is an underestimate, given that 18% of the initially chosen subjects were too ill to take part in the MMSE assessment and the relatively low sensitivity of the assessment [62]. Burra et al. [63] found that the prevalence of cognitive deficits in the homeless population as assessed by MMSE ranged from 4% to 7%. Backer and Howard [64] discussed possible sources of cognitive impairment, including schizophrenia, substance abuse, traumatic or acquired brain injury and developmental disabilities. They pointed out that cognitive impairment in the homeless is often missed, as service workers more often focus on survival needs.

In the present study, only four of the subjects with dementia or cognitive impairment had been given a previous psychiatric assessment for cognitive impairment. The presence and consequences of cognitive impairment were often overlooked by service providers. The cognitively impaired subjects were usually the quiet and submissive ones, who had no history of violence or self-harm and no active complaints. They were the group who were prone to be abused.

#### Psychotic disorders

The prevalence of psychotic disorders in the subjects was 10%. This figure is an underestimate, as a group of subjects who had evidence of psychotic symptoms were not included in the data analysis.

Folsom and Jeste [33], in their review of the prevalence of schizophrenia in homeless people, reported a prevalence of 11%. This figure might also be an underestimate, as only 7 of the 33 reviewed studies included subjects living on the street.

A study in the United States [65] found that 15% of the 237 patients with psychotic disorders had experienced at least one episode of homelessness before or within 24 months of their first psychiatric admission. In subjects with schizophrenia and related disorders, those with a high level of negative symptoms had a significantly higher risk of pre-hospitalisation homelessness than those with a low symptom level. The study postulated that homelessness itself could be a cause of mental illness, or it could occur in the prodromal phase of psychotic disorders. Odell and Commander [66], in their study of 39 pairs of matched case control homeless and never -homeless subjects with psychotic disorders, found that the homeless subjects were more likely to have lost contact with childhood carers, and to have experienced drug and alcohol abuse, arrests, convictions and imprisonments.

In the present study, a significant number of homeless people with psychotic symptoms were too ill to complete the assessment. Their mental illness rendered them unable to give valid consent. Some of these individuals might be mentally -incapacitated. Apart from evidence of psychotic symptoms, they also showed severe self-neglect. Most of them had not lived in homeless shelters before. They also refused to receive any medical treatment or psychiatric assessment. The appropriate use of compulsory treatment should be reinforced to protect this group of untreated psychotic patients who lack the ability to take care of their basic needs.

### Barriers to Accessing Psychiatric Services

In the current study, only 41% of the mentally ill subjects had ever had any psychiatric assessment, and only 13% were receiving treatment at the time of interview. The true figures would be even lower as a significant proportion of the severely ill subjects were not included in analysis. These figures suggest that barriers to mental health services exist. Two -third of the subjects who had reported mental health problems in the past thought there was no need to seek medical assessment and the remainder preferred self-help to medical consultation

Kim et al. [67] suggested that perceived stigma was an important barrier for accessing mental health service in those with serious mental illness. In a study with more than 5000 subjects with serious mental illness, Kessler et al. [68] found out that situational barriers, financial barriers and perceived lack of effectiveness were the main reasons for not seeking medical treatment. Homeless people solving the problem on their own was the most commonly reported reason for failing to seek treatment. Other barriers to accessing health care services include the presence of psychiatric symptoms, personal barriers [69], housing instability, food insecurity [70], frequent subsistence difficulty [71], and previous negative experience of mental health services [5]. In Hong Kong, where the public health care service is practically free, the individual’s perception of his or her problem is probably the most important factor, and it can only be resolved by establishing trusting relationships between the care workers and their clients.

The findings of the current study also suggest that the local health care system might have intrinsic barriers that inhibit the provision of timely and appropriate mental health services for homeless people in Hong Kong. The existence of the 17 severely mentally ill subjects who had to be excluded from the study is a strong proof of this. They were mentally incapacitated, had no insight into their problem and rejected any offer made by care workers apart from those directly relating to survival. The only way to secure proper psychiatric or medical treatment is through compulsory procedures under the local Mental Health Ordinance.

The Mental Health Ordinance [72] governs the care, supervision, detention and treatment of mentally incapacitated and mentally disordered persons. The Ordinance grants registered doctors the power to apply for guardianship of mentally incapacitated person, to provide medical treatment and to arrange compulsory psychiatric treatment for them. Under this ordinance, mentally ill persons can be detained in the interests of his own health or safety, or with a view to the protection of other persons. Interpreting ‘his safety’ and ‘the protection of other persons’ is straightforward, but ‘in the interests of his own health’ is too broad and inclusive and will inevitably invite challenges. It is thus seldom invoked and is often forgotten or neglected. As a result, the health care or social workers often excuse themselves from taking a more active role in tackling the mental problem of homeless subjects unless there is a risk of imminent violence or self-harm. Large, Nielssen, Ryan and Hayes [73] investigated the relationship between the duration of untreated psychosis and mental health laws. They concluded that mental health laws that require the patients to be assessed as dangerous before they could receive involuntary treatment were associated with a significantly longer duration of untreated psychosis. It is thus recommended that mental health care professionals and social workers should be better informed about and trained in aspects of compulsory treatment and the Mental Health Ordinance for the benefit of their clients.

The compulsory treatment order under the Mental Health Ordinance has a limited period of validity. Once a patient has recovered, the patient is no longer a mentally incapacitated person. The patient can no longer be kept in mental hospital if this is against his or her will. A variety of services are available to help the patient to integrate into the community. For example, the day hospital and the supported employment service aim to train patients to work again. The supported hostel and halfway house provide all-rounded services, from self-care training, budgeting, drug supervision to communication skills and job training. It is hoped that after leaving the supported hostel or halfway house, patients can lead an independent life.

Even equipped with a good understanding of the Mental Health Ordinance, deciding when and where intervention should take place, and balancing patient autonomy with the principle of beneficence generally requires much skill and experience. In addition, homeless people usually move around during the day and return at night to a sleeping place, which may be outdoors, and could change frequently. Finding them at the right time and place taxes the efficiency and flexibility of community outreach teams, which only provide outreach services during office hours. Any rigid policy to see clients only at a designated place and time would exclude them from effective assessment. Close collaboration between the community psychiatric team and social workers with flexible tactics is therefore essential if comprehensive mental health coverage for homeless people is to be secured.

### Study Strengths and Limitations

The current study presents the first systematic face-to-face field survey of mental problems in homeless people by a trained psychiatrist. About 100 successful assessments were made with standard diagnostic tools through live interviews that were conducted mainly at night, either at social centers or in the street. The quality of the diagnosis was held up to the highest standard possible. Meticulous effort was also made to identify and trace the subjects.

Despite all of the efforts made, limitations abound. This study sampled subjects from identifiable groups of homeless people. The characteristics of the unidentified group and the untraceable group are not known. Eighteen per cent of the chosen subjects who could not complete the SCID or give valid consent were excluded from the analysis. Thus, although the stringent methodology obtained reliable results, there was a substantial loss of subjects representing the severe end of the spectrum of mental disability. The results might therefore only reflect the condition of subjects with better functioning. The prevalence of intellectual disability is likely to be grossly underestimated, as the study did not include any standardised assessment for intellectual disability, which is a neglected area in the field [61]. Lastly, only Chinese homeless individuals were recruited to the study. The mental health characteristics of homeless ethnic minority and refugee population remain unknown.

## Conclusion

This is the first study in Hong Kong to use standardised diagnostic instruments to investigate the prevalence of mental illness among homeless individuals. The study found a high prevalence of mental illness among the homeless population. Moreover, the most severely ill subjects were not included in detailed analysis due to methodological design. They are the most needy but their needs have often been neglected. The presence of this untreated and unreached severely mentally ill group of homeless people, and the low treatment rate, indicates that the mental health care system is failing to provide proper care for the most seriously ill people on the street. Close collaboration between mental health care workers and social workers, together with appropriate use of compulsory treatment and flexible tactics, would benefit this subgroup of homeless people who survive at the lowest stratum of society.

## References

[1] Crisis. How many and how much: Single homelessness and the question of number and cost. London: Crisis UK 2003.

[2] National Alliance to End Homelessness. State of homelessness. 2012. Available: http://www.endhomelessness.org/library/entry/soh-2012-chapter-one-homelessness-counts.

[3] Legislative Council. Background brief prepared by the Legislative Council Secretariat for the joint meeting on 13 April 2012 Support services for street sleepers. 2012. Available: http://www.legco.gov.hk/yr11-12/english/panels/ws/papers/hgws0413cb2-1601-2-e.pdf.

[4] St. James Settlement & Richmond Fellowship of Hong Kong. Lo suk je jing san gin hong yin gau bo go. [Survey of the mental health of street sleepers in Hong Kong]. 2010. Hong Kong.

[5] Scott J. Homelessness and mental illness. Br J Psychiatry. 1993; 162:314–324. 8453425 10.1192/bjp.162.3.314

[6] D’Amore J, Hung O, Chiang W, Goldfrank L. The epidemiology of the homeless population and its impact on an urban emergency department. Acad Emerg Med. 2001; 8:1051 11691667 10.1111/j.1553-2712.2001.tb01114.x

[7] Yamanaka K, Kondo T, Miyao M. Tuberculosis among the homeless people. Respir Med. 1994;88: 763–769. 7846338 10.1016/s0954-6111(05)80199-4

[8] Hibbs JR, Benner L, Klugman L, Spence R, Macchia I, Mellinger A, et al Mortality in a cohort of homeless adults in Philadelphia. N Engl J Med. 1994; 331(5):304–309. 8022442 10.1056/NEJM199408043310506

[9] Nielsen SF, Hjorthøj CR, Erlangsen A, Nordentoft M. Psychiatric disorders and mortality among people in homeless shelters in Denmark: a nationwide register-based cohort study. Lancet. 2011;377: 2205–2214. 10.1016/S0140-6736(11)60747-2 21676456

[10] Barrow SM, Herman DB, Córdova P, Struening EL. Mortality among homeless shelter residents in New York City. Am J Public Health. 1999; 89:529–534. 10191796 10.2105/ajph.89.4.529 PMC1508869

[11] Hwang SW, Orav EJ, O’Connell JJ, Lebow JM, Brennan TA. Causes of death in homeless adults in Boston. Ann Intern Med.1997; 126:625–628. 9103130 10.7326/0003-4819-126-8-199704150-00007

[12] Centers for Disease Control and Prevention. Deaths among homeless persons, San Francisco, 1985–1990. Morbidity and Mortality Weekly Report. 1991; 40(50):877–880. 1749371

[13] Communities and Local Government. More than a roof: a report into tackling homelessness. London:UK. 2003. Available: http://www.communities.gov.uk/documents/housing/pdf/156600.pdf.

[14] Koegel P, Melamid E, Burnam MA. Childhood risk factors for homelessness among homeless adults. Am J Public Health. 1995; 85:1642–1649. 7503338 10.2105/ajph.85.12.1642 PMC1615728

[15] Shelton KH, Taylor PJ, Bonner A, Bree Mvd. Risk factors for homelessness: Evidence from a population-based study. Psychiatr Serv. 2009; 60:465–472. 19339321 10.1176/ps.2009.60.4.465

[16] Davies-Netzley S, Hurlburt MS, Hough RL. Childhood abuse as precursor to homelessness for homeless women with severe mental illness. Violence Vict. 1996; 11(2):129–142. 8933709

[17] Herman DB, Susser ES, Struening EL, Link BL. Adverse childhood experiences: Are they risk factors for adult homelessness? Am J Public Health. 1997; 87:249–255. 9103105 10.2105/ajph.87.2.249 PMC1380802

[18] O’Toole TP, Gibbon JL, Hanusa BH, Fine MJ. Utilization of health care resources among subgroups of urban homeless and housed poor. J Health Polit Policy Law. 1999; 24(1):91–114. 10342256 10.1215/03616878-24-1-91

[19] Connell MJ, Kasprow W, Rosenheck RA. Rates and risk factors for homelessness after successful housing in a sample of formerly homeless veterans. Psychiatr Serv. 2008; 59(3):269–275. 10.1176/ps.2008.59.3.268 18308907

[20] Goldfinger SM, Schutt RK, Tolomiczenko GS, Seidman L, Penk WE, Turner W, et al Housing placement and subsequent days homeless among formerly homeless adults with mental illness. Psychiatr Serv. 1999; 50(5): 674–679. 10332905 10.1176/ps.50.5.674

[21] Henry JM, Boyer L, Belzeaux R, Baumstarck-Barrau K, Samuelian JC. Mental disorders among homeless people admitted to a French psychiatric emergency service. Psychiatr Serv. 2010; 61(3):264–271. 20194403 10.1176/ps.2010.61.3.264

[22] Kuno E, Rothbard AB, Averyt J, Culhane D. Homelessness among persons with serious mental illness in an enhanced community-based mental health system. Psychiatr Serv. 2000; 51(8):1012–1016. 10913454 10.1176/appi.ps.51.8.1012

[23] Riesdorph-Ostrow W. Deinstitutionalization: A public policy perspective. J Psychosoc Nurs Ment Health Serv. 1989; 27(6):4–8. 2738866 10.3928/0279-3695-19890601-04

[24] Taylor G. Bitter Freedom: Deinstitutionalization and the Homeless. J. Contemp. Health L. & Pol'y. 1987; 3:205. 10282310

[25] Kushel MB, Vittinghoff E, Haas JS. Factors associated with the health care utilization of homeless persons. JAMA. 2001; 285:200–206. 11176814 10.1001/jama.285.2.200

[26] Frazier SH. Responding to the needs of the homeless mentally ill. Public Health Rep. 1985; 100(5): 462–469. 3931159 PMC1425074

[27] Lamb HR, Bachrach LL. Some perspectives on deinstitutionalization. Psychiatr Serv. 2001; 52(8): 1039–1045. 11474048 10.1176/appi.ps.52.8.1039

[28] Lamb HR, Weinberger LE. Persons with severe mental illness in jails and prisons: A review. Psychiatr Serv. 1998; 49(4): 483–492. 9550238 10.1176/ps.49.4.483

[29] Aderibigbe YA. Deinstitutionalization and criminalization: Tinkering in the interstices. Forensic Sci Int. 1997; 85: 127–134. 9061906 10.1016/s0379-0738(96)02087-7

[30] Cohen CI, Thompson KS. Homeless mentally ill or mentally ill homeless? Am J Psychiatry. 1992; 149(6):816–823. 1590500 10.1176/ajp.149.6.816

[31] Fazel S, Khosla V, Doll H, Geddes J. The prevalence of mental disorders among the homeless in Western countries: systematic review and meta-regression analysis. PLoS Med. 2008; 5(12):225 Available: http://www.plosmedicine.org/article/info%3Adoi%2F10.1371%2Fjournal.pmed.0050225. 10.1371/journal.pmed.0050225 PMC2592351 19053169

[32] Reardon ML, Burns AB, Preist R, Sachs-Ericsson N, Lang AR (2003) Alcohol use and other psychiatric disorders in the formerly homeless and never homeless: prevalence, age of onset, comorbidity, temporal sequencing, and service utilization. Subst Use Misuse. 2003; 38:601–644. 12747399 10.1081/ja-120017387

[33] Folsom D, Jeste DV (2002) Schizophrenia in homeless persons: a systematic review of the literature. Acta Psychiatr Scand. 2002; 105: 404–413. 12059843 10.1034/j.1600-0447.2002.02209.x

[34] Prigerson HG, Desai RA, Liu- Mares W, Rosenheck RA. Suicidal ideation and suicide attempts in homeless mentally ill persons. Soc Psychiatry Psychiatr Epidemiol. 2003; 38: 213–219. 12664232 10.1007/s00127-003-0621-8

[35] Sullivan G, Burnam A, Koegel P, Hollenberg J. Quality of life of homeless persons with mental illness: results from the course-of- homelessness study. Psychiatr Serv. 2000; 55:1135–1141. 10.1176/appi.ps.51.9.1135 10970916

[36] White MC, Chafetz L, Collins-Bride G, Nickens J. History of arrest, incarceration and victimization in community-based severely mentally ill. J Community Health. 2006; 31: 123–135. 16737173 10.1007/s10900-005-9005-1

[37] Maniglio R. Severe mental illness and criminal victimization: A systematic review. Acta Psychiatr Scand. 2009;119:180–191. 10.1111/j.1600-0447.2008.01300.x 19016668

[38] Fichter MM, Koniarczyk M, Greifenhagen A, Koegel P, Quadflieg N, Wittchen HU, et al Mental illness in a representative sample of homeless men in Munich, Germany. Eur Arch Psychiatry Clin Neurosci. 1996; 246: 185–196. 8832196 10.1007/BF02188952

[39] Han OS, Lee HB, Ahn JH, Park JI, Cho MJ, Hong JP, et al Lifetime and current prevalence of mental disorders among homeless men in Korea. J Nerv Ment Dis., 2003; 191(4): 272–275. 12695742 10.1097/01.NMD.0000061151.19363.82

[40] Iachan R, Dennis ML. A multiple frame approach to sampling the homeless and transient population. J Off Stat. 1993; 9: 747–764.

[41] Cordray DS, Pion GM. What’s behind the numbers? Definitional issues in counting the homeless Understanding homelessness: New Policy and Research Perspectives. Washington DC: Fannie Mae Foundation, 1997.

[42] National Coalition for the Homeless. How many people experience homelessness? 2007. Available: http://www.nationalhomeless.org/publications/facts/How_Many.pdf.

[43] Fischer PJ, Breakey WR. The epidemiology of alcohol, drug and mental disorders among homeless persons. Am Psychol. 1991; 46: 1115–1128. 1772149 10.1037//0003-066x.46.11.1115

[44] Gill B, Meltzer H, Hinds K. The prevalence of psychiatric comorbidity among homeless adults. Int Rev Psychiatry. 2003; 15:134–140. 12745321 10.1080/0954026021000046056

[45] Hwang SW. Mental illness and mortality among homeless people. Acta Psychiatr Scand. 2001; 103(2): 81–82. 11167309 10.1034/j.1600-0447.2001.103002081.x

[46] Social Welfare Department. Services for Street Sleepers. 2012. Available: http://www.swd.gov.hk/en/index/site_pubsvc/page_family/sub_listofserv/id_serstsleeper/.

[47] Society for Community Organization. Hong Kong lo suk jie diu cha bo go. [Survey of the homeless in Hong Kong]. 2010. Hong Kong.

[48] Legislative Council. Panel on Welfare Services. Minutes of meeting held on 9 May 2011. 2011. Available: http://www.legco.gov.hk/yr10-11/english/panels/ws/minutes/ws20110509.pdf.

[49] Scheaffer RL, Mendenhall IW, Ott RL, Gerow KG. Elementary survey sampling. Boston: Cengage Learning; 2011.

[50] Pedersen G, Karterud S. The symptom and function dimensions of the Global Assessment of Functioning (GAF) scale. Compr Psychiatry. 2012; 53(3): 292–298. 21632038 10.1016/j.comppsych.2011.04.007

[51] Chiu HF, Lam LC, Chi I, Leung T, Li SW, Law WT, et al Prevalence of dementia in Chinese elderly in Hong Kong. Neurology. 1998; 50(4): 1002–1009. 9566386 10.1212/wnl.50.4.1002

[52] Rossi P. Down and out in America: The origins of homelessness. Chicago IL: University of Chicago Press 1989.

[53] Schutt R, Meschede T, Rierden J. Distress, suicidal thoughts and social support and homeless adults. J Health Soc Behav. 1994; 35: 134–142. 8064121

[54] Irwin J, Lagory M, Ritchey F, Fitzpatrick K. Social assets and mental distress among the homeless: Exploring the roles of social support and other forms of social capital on depression. Soc Sci Med. 2008; 67: 1935–1943. 10.1016/j.socscimed.2008.09.008 18930571

[55] Ritchey FJ, Lagory M, Fitzpatrick KM, Mullis J. A comparison of homeless, community-wide, and selected distressed samples on the CES-Depression Scale. Am J Public Health, 1990; 80: 1384–1386. 2240314 10.2105/ajph.80.11.1384 PMC1404896

[56] Vázquez C, Muñoz M, Sanz J. Lifetime and 12-month prevalence of DSM-III-R mental disorders among the homeless in Madrid: A European study using the CIDI. Acta Psychiatr Scand. 1997; 95(6): 523–530. 9242848 10.1111/j.1600-0447.1997.tb10141.x

[57] Gottlieb LE. The health care of homeless persons- Part IV: Depression. Boston: Boston Health Care for the Homeless Program. 2004. Available: http://www.bhchp.org/BHCHP%20Manual/pdf_files/Part4_PDF/Depression.pdf.

[58] Department of Health. Alcohol and health: Hong Kong situation. Hong Kong: China The Government of the Hong Kong Special Administrative Region of the People’s Republic of China Available: http://www.dh.gov.hk/english/pub_rec/pub_rec_ar/pdf/ncd_ap2/action_plan_2_alcohol%20and%20health%20HK%20situation_e.pdf.

[59] Gilchrist G, Morrison DS. Prevalence of alcohol-related brain damage among homeless hostel dwellers in Glasgow. Eur J Public Health. 2005; 15(6):587–588. 16162595 10.1093/eurpub/cki036

[60] Smith I, Hillman A. Management of Alcohol Korsakoff Syndrome. Adv Psychiatr Treat. 1999; 5: 271–278.

[61] Fitzpatrick S, Kemp P, Klinker S. Single homelessness: an overview of research in Britain. Bristol: Policy Press 2000 Available: http://www.jrf.org.uk/publications/single-homelessness-research-overview.

[62] Gonzalez EA, Dieter JN, Natale RA, Tanner SL. Neuropsychological evaluation of higher functioning homeless persons: A comparison of an abbreviated test battery to the mini-mental state exam. J Nerv Ment Dis. 2001; 189(3):176–181. 11277354 10.1097/00005053-200103000-00006

[63] Burra TA, Stergiopoulos V, Rourke SB. A systematic review of cognitive deficits in homeless adults: implications for service delivery. Can J Psychiatry. 2009; 54(2):123–133. 19254443 10.1177/070674370905400210

[64] Backer TE, Howard EA. Cognitive impairments and the prevention of homelessness: Research and practice review. J Prim Prev. 2007; 28(3–4): 375–388. 17549638 10.1007/s10935-007-0100-1

[65] Herman DB, Susser ES, Jandorf PHL, Lavelle J, Bromet EJ. Homelessness among individuals with psychotic disorders hospitalized for the first time: Findings from the Suffolk County Mental Health Project. Am J Psychiatry. 1998; 155:109–113. 9433347 10.1176/ajp.155.1.109

[66] Odell SM, Commandar MJ. Risk factors for homelessness among people with psychotic disorders. Soc Psychiatry Psychiatr Epidemiol. 2000; 35:396–401. 11089667 10.1007/s001270050256

[67] Kim MM, Swanson JW, Swartz MS, Bradford DW, Mustillo SA, Elbogen EB. Health care barriers among severely mentally ill homeless adults: Evidence from a five-site health and risk study. Adm Policy Ment Health. 2007; 34:363–375. 17294124 10.1007/s10488-007-0115-1

[68] Kessler RC, Berglund PA, Bruce ML, Koch JR, Laska EM, Leaf PJ, et al The prevalence and correlates of untreated serious mental illness. Health Serv Res. 2001; 36:987–1007. 11775672 PMC1089274

[69] Drapalski AL, Milford J, Goldberg RW, Brown CH, Dixon LB. Perceived barriers to medical care and mental health care among veterans with serious mental illness. Psychiatr Serv. 2008; 59(8):921–924. 10.1176/appi.ps.59.8.921 18678691

[70] Kushel MB, Gupta R, Gee L, Haas JS. Housing instability and food insecurity as barriers to health care among low-income Americans. JGIM. 2006; 21(1):71–77. 16423128 10.1111/j.1525-1497.2005.00278.x PMC1484604

[71] Gelberg L, Gallagher TC, Andersen RM, Koegel P. Competing priorities as a barrier to medical care among homeless adults in Los Angeles. Am J Public Health. 1997; 87(2): 217–220. 9103100 10.2105/ajph.87.2.217 PMC1380797

[72] Department of Justice. Mental health ordinance. Hong Kong: China The Government of the Hong Kong Special Administrative Region of the People’s Republic of China 1999 Available: http://www.legislation.gov.hk/blis_pdf.nsf/6799165D2FEE3FA94825755E0033E532/4D4C0652AC60B789482575EE00433474/$FILE/CAP_136_e_b5.pdf.

[73] Large MM, Nielssen O, Ryan CJ, Hayes R. Mental health laws that require dangerousness for involuntary admission may delay the initial treatment for schizophrenia. Soc Psychiatry Psychiatr Epidemiol. 2007; 43: 251–256. 18060340 10.1007/s00127-007-0287-8

